# The Effect of Cromolyn Sodium and Nedocromil Sodium Administered by A pressurized Aerosol with A spacer Device on Exercise-Induced Asthma in Children

**DOI:** 10.1155/S0962935194000736

**Published:** 1994

**Authors:** F. M. de Benedictis, G. Tuteri, A. Niccoli, D. Mezzetti, L. Rossi, L. Bruni

**Affiliations:** 1 Clinica Pediatrica Policlinico Monteluce Università di Perugia Perugia 06100 Italy

## Abstract

To compare the effectiveness of cromolyn sodium (CS) (10 mg) and nedocromil sodium (NS) (4 mg) administered by a metered dose inhaler (MDI) with a spacer device in preventing exercise-induced asthma (EIA), eight asthmatic children with EIA were studied in a randomized double-blind, cross-over, placebo-controlled study, CS and NS provided significant, comparable protection from EIA and both were better than placebo. We conclude that CS and NS administered by a pressurized aerosol with a spacer device provide equal protection against EIA in children.


Mediators of Inflammation 3, S35–S37 (1994)

MOIa10

### The effect of cromolyn sodium and nedocromil sodium administered by a pressurized aerosol with a spacer device on exercise-induced asthma in children

**To compare the effectiveness of cromolyn sodium (CS) (10 mg) and nedocromil sodium (NS) (4 mg) administered by a metered dose inhaler (MDI) with a spacer device in preventing exercise-induced asthma (EIA), eight asthmatic children with EIA were studied in a randomized double-blind, cross-over, placebo-controlled study. CS and NS provided significant, comparable protection from EIA and both were better than placebo. We conclude that CS and NS administered by a pressurized aerosol with a spacer device provide equal protection against EIA in children.**

**Key words:** Children, Cromolyn sodium, Exercise-induced asthma, Nedocromil sodium

**F. M. de Benedictis,[CA] G. Tuteri, A. Niccoli, D. Mezzetti, L. Rossi and L. Bruni**

Clinica Pediatrica, Policlinico Monteluce, Università di Perugia, 06100 Perugia, Italy

[CA] Corresponding Author

### Introduction

Cromolyn sodium (CS) and nedocromil sodium (NS) are two anti-inflammatory drugs which have been shown to be effective in preventing exercise-induced asthma (EIA) in both adults and children.[1–4] Metered dose inhalers provide an aerosolized dose with high particle velocity, necessitating respiratory coordination to obtain optimal drug deposition in the lung. The use of a spacer device attached to the MDI reduces the velocity of aerosol particles and significantly improves drug delivery to the peripheral lung.[5] To compare the effectiveness of CS and NS administered by a MDI with a spacer device in preventing EIA in childhood, a double-blind, cross-over, placebo-controlled study was performed.

### Subjects and Methods

The study was double-blind, randomized, cross-over and placebo-controlled. Eight patients (five males, three females), aged 7 to 11 years (mean 8.7 ± 11.2 years) were recruited. All patients attended the Pediatric Asthma Clinic at Perugia General Hospital, and all had asthma as defined by the American Thoracic Society.[6] All subjects previously demonstrated a consistent fall in $FEV_1$ of at least 15% from baseline after a 6 min standard treadmill exercise screening test. They were being treated with different anti-asthmatic regimens, such as sustained release theophylline, beta-agonists, SCG, NCS, and inhaled steroids; none was under therapy with oral steroids. Sustained release theophylline was withheld for 24 h, and other drugs for 12 h before each exercise test. None of the subjects had had respiratory infections in the 4 weeks before the trial. Informed consent was obtained from patients and their parents, and the protocol was approved by the Hospital Ethics Committee.

The screening test consisted of steady state running for 6 min on a treadmill at an incline which would produce a heart rate of at least 85% of the maximum predicted for age.[7] After screening in randomized order on 3 separate days, patients were tested on different treatments inhaled from a metered dose inhaler with a spacer device (Aerochamber, Trudell Medical, London, Ontario): SCG (5 mg twice), or NCS (2 mg twice), or placebo (2 puffs). The drugs were administered by a trained physician, and all the patients were skilled in the use of MDI with aerochamber. The patients performed the exercise test 20 min after every drug inhalation. Each patient always performed tests at the same time of the day, and all four tests were completed within 10 days.

Room temperature and relative humidity were monitored. Differences of 1°C in temperature and 5 mg $H_2O$/l of air in water content on the test days of each patient were considered acceptable.[8] Room temperatures ranged from 21 to 23°C, and relative humidity from 48% to 58% on the different study days.

Pulmonary function was measured by a turbin spirometer (Pocket Spirometer I: Micro Medical Limited, Rochester, Kent, UK), according to accepted standards.[9,10] Predicted normal values for spirometry were obtained from the study of Knudson *et al.*[11] All children were already familiar with the spirometer. Measurements were performed before drug inhalation (baseline value), before every exercise test (pre-

© 1994 Rapid Communications of Oxford Ltd exercise value), and then 3, 5, 10, 15 and 30 min after the end of exercise. Heart rate was also measured before and immediately after the exercise. The exercise test was performed only if the baseline $FEV_1$ was greater than 70% of the mean predicted for the child's height, and if the baseline $FEV_1$ varied < 10% from the values on previous test days. The following indices were calculated from the results of the pulmonary function tests:

(A) Maximum % fall $FEV_1$

$$= \frac{\text{pre-exercise } FEV_1 - \text{lowest } FEV_1 \text{ post-exercise}}{\text{pre-exercise } FEV_1}$$

$$\text{(B) \% protection } FEV_1 = \frac{Ps - Pt}{Ps}$$

where Ps is the percentage fall $FEV_1$ at the screening test, and Pt is the percentage fall $FEV_1$ after each treatment.

Complete protection was considered to have been obtained if the percentage fall in $FEV_1$ was within the normal range (< 10%).[12] Clinical protection was considered to have been obtained if the percentage fall after receiving the active drug was half or less of the percentage fall after receiving placebo.

*Analysis of data:* Analysis of variance for repeated measures and Student's *t*-test for paired data, including the Bonferroni adjustment, were used. Differences were considered significant if $p < 0.05$.

### Results

Mean pre-drug baseline $FEV_1$ values on different study days were statistically comparable (CS: 1.60 L; NS: 1.62 L; PL: 1.57 L), and no change of mean $FEV_1$ was observed 20 min after administration of each one of three formulations (CS: 1.58 L; NS: 1.63 L; PL: 1.61 L). No statistically significant differences emerged in pre-exercise values in the three groups.

The mean maximum percentage fall in $FEV_1$ (± S.D.) in the screening test, after placebo, CS and NS was 38.8 ± 11.2, 31.4 ± 20.6, 14.8 ± 18.6 and 13.3 ± 8.1. A significant decrease in mean percentage fall in $FEV_1$ with respect to baseline exercise test was observed after treatment with CS and NS, but not with placebo. Student *t*-test baseline versus: (A) Placebo: $p$ = not significant; (B) CS: $p < 0.005$; (C) NS: $p < 0.005$. The decrease of percentage fall $FEV_1$ obtained with CS and NS was comparable (Student *t*-test: $p$ = not significant), and both drugs were significantly better than placebo ($p < 0.005$). It was found that 5/8 (62.5%), 4/8 (50%) and 1/8 (12.5%) subjects were completely protected by CS, NS and placebo, respectively. Only 4/8 (50%) patients received complete protection from both active drugs (Table 1).

**Table 1.** Maximum percentage fall in $FEV_1$ after exercise

| Patient *n* | Screening | Placebo | CS | NS |
|---|---|---|---|---|
| 1 | 40.2 | 22.1 | 20.1 | 6.3 |
| 2 | 61.4 | 35.3 | 15.9 | 32.7 |
| 3 | 28.0 | 1.3 | 10.0 | 0.6 |
| 4 | 36.0 | 18.0 | 9.9 | 5.6 |
| 5 | 45.3 | 52.9 | 16.9 | 17.1 |
| 6 | 38.6 | 67.4 | 28.6 | 52.3 |
| 7 | 36.8 | 24.7 | 0.0 | 0.0 |
| 8 | 24.8 | 29.5 | 5.1 | 3.8 |
| Mean | 38.8 | 31.4 | 13.3 | 14.8 |
| SD | 11.2 | 20.6 | 8.9 | 18.6 |

**Table 2.** Percentage of protection from EIA

| Patient *n* | Placebo | CS | NS |
|---|---|---|---|
| 1 | 45 | 84 | 50 |
| 2 | 42 | 47 | 74 |
| 3 | 95 | 98 | 64 |
| 4 | 49 | 84 | 72 |
| 5 | 0 | 62 | 62 |
| 6 | 0 | 0 | 26 |
| 7 | 32 | 100 | 100 |
| 8 | 0 | 85 | 79 |
| Mean | 32.8 | 13.3 | 14.8 |
| SD | 32.9 | 8.9 | 18.6 |

The protection percentage was 70.0 ± 33.3, 65.8 ± 21.7 and 32.8 ± 32.9 for CS, NS and placebo, respectively. A protection value greater than 50% (clinical protection) was obtained in 6/8 (75%) patients treated with SC, 7/8 (87.5%) treated with NS, and 1/8 (12.5%) patients who received placebo (Table 2).

### Discussion

Both CS and NS have been shown to be effective in protecting against EIA in children.[3,4] The mechanism by which these two drugs exert their action in preventing EIA has yet to be determined, but both drugs exhibit a considerable protective effect on the mucosal mast cells *in vivo* and *in vitro*.[13,14]

This double-blind, within patient comparative study shows that CS and NS inhaled by a MDI with a spacer device have a significant and comparable effect in preventing EIA in children, and that both drugs are more effective than placebo.

Our data are in agreement with those from other studies, which showed the comparable effectiveness of CS and NS in preventing EIA, both in adults[15,16] and in children.[17,18] A variable protective effect, which is highlighted by the large standard deviation of the mean maximum fall in $FEV_1$, was found between CS and NS in some of our patients. This has been previously reported in other studies[16,19] and may reflect the response variability that exists between asthmatics.[15]

Patel and Albazzaz[19] found a significantly higher protection against EIA with NS when compared with CS. In addition, Morton *et al.*[15] reported that the percentage of adults who were completely protected from EIA was higher after NS (62.5%) than after CS (25%). However, as CS was administered at a low dosage, the results may have been distorted.

The use of a spacer device in adjunct to MDI has been strongly advocated for children who may not be able to perform the inhalation correctly.[5] However, although spacer devices have several potential advantages over MDIs, their use did not modify the effect of both CS and NS in a previous study.[17] This was probably due to the correct technique by which previously skilled children used MDI, allowing optimal drug deposition to peripheral airways.

A statistically significant effect of a drug on EIA does not necessarily indicate the effect is clinically important. Therefore, we also evaluated the protection percentage against EIA, as an index of a good clinical control. This analysis yielded the same results as the analysis of the percentage fall $FEV_1$ itself.

It is concluded that CS and NS provide equal protection against EIA in children when the clinical recommended dose is administered by a pressurized aerosol with an aerochamber. Further studies are needed to compare the duration of action of these two drugs in inhibiting EIA.

### References

1. Patel KR, Wall RT. Dose-duration effect of sodium cromoglycate aerosol in exercise-induced asthma. *Eur J Respir Dis* 1986; **69**: 256–260.
2. Albazzaz MK, Neale MJ, Patel KR. Dose-response study of nebulized nedocromil sodium in exercise-induced asthma. *Thorax* 1989; **44**: 816–819.
3. Corkey C, Mindorff C, Levison H, Newth C. Comparison of three different preparations of disodium cromoglycate in the prevention of exercise-induced bronchospasm. *Am Rev Respir Dis* 1982; **125**: 623–626.
4. Chudry N, Correa F, Silverman M. Nedocromil sodium and exercise induced asthma. *Arch Dis Child* 1987; **62**: 412–414.
5. Levison H, Reilly PA, Worsley GH. Spacing devices and metered dose inhalers in childhood asthma. *J Pediatr* 1985; **107**: 662–668.
6. American Thoracic Society. Chronic bronchitis, asthma, and pulmonary emphysema. *Am Rev Respir Dis* 1962; **85**: 762–768.
7. Egglestone PA, Rosenthal RR, Anderson SA, *et al.* Guidelines for the methodology of exercise challenge testing of asthma. *J Allergy Clin Immunol* 1979; **64**: 642–646.
8. Anderson SD, Schoeffel RE, Follet R, *et al.* Sensitivity to heat and water loss at rest and during exercise in asthmatic patients. *Eur J Respir Dis* 1982; **63**: 459–463.
9. Taussig LM, Chernick V, Wood R, *et al.* Standardization of lung function testing in children. *J Pediatr* 1980; **97**: 668–675.
10. American Thoracic Society Statement. Snowbird workshop on standardization of spirometry. *Am Rev Respir Dis* 1979; **119**: 831–838.
11. Knudson RJ, Slatin RC, Lebowitz MD, *et al.* The maximal expiratory flow volume curve. *Am Rev Respir Dis* 1976; **113**: 587–592.
12. Silverman M, Anderson SD. Standardization of exercise tests in asthmatic children. *Arch Dis Child* 1972; **47**: 882–887.
13. Eady RP, Greenwood B, Jackson DM, *et al.* The effect of nedocromil sodium and sodium cromoglycate on antigen induced bronchoconstriction in the Ascaris-sensitive monkey. *Br J Pharmacol* 1985; **85**: 323–327.
14. Leung KBP, Flint KC, Brostoff J, *et al.* A comparison of nedocromil sodium and sodium cromoglycate on human lung mast cells obtained by bronchoalveolar lavage and by dispersion of lung fragments. *Eur J Respir Dis* 1986; **69** (Suppl 147): 223–226.
15. Morton AR, Ogle SL, Fitch KD. Effects of nedocromil sodium, cromolyn sodium and placebo in exercise-induced asthma. *Ann Allergy* 1992; **68**: 143–148.
16. König P, Hordvik NI, Kreutz C. The preventive effect and duration of action of nedocromil sodium and cromolyn sodium on exercise induced asthma (EIA) in adults. *J Allergy Clin Immunol* 1987; **79**: 64–68.
17. Comis A, Valletta EA, Sette L, *et al.* Comparison of nedocromil sodium and sodium cromoglycate administered by pressurized aerosol, with and without a spacer device in exercise-induced asthma in children. *Eur Respir J* 1993; **6**: 523–526.
18. Novembre E, Veneruso G, Frangia G, *et al.* Inhibition of exercise-induced asthma by nedocromil sodium and cromoglycate in children. *J Allergy Clin Immunol* 1993; **91**: 168 (abstract).
19. Patel KL, Albazzaz MK. Protective effect of cromolyn sodium and nedocromil sodium in exercise-induced asthma. *J Allergy Clin Immunol* 1987; **79**: 187 (abstract).

## References

[PDF_00239] Albazzaz M. K., Neale M. G., Patel K. R. (1989). Dose-response study of nebulised nedocromil sodium in exercise induced asthma.. Thorax.

[PDF_00252] Anderson S. D., Schoeffel R. E., Follet R., Perry C. P., Daviskas E., Kendall M. (1982). Sensitivity to heat and water loss at rest and during exercise in asthmatic patients.. Eur J Respir Dis.

[PDF_00244] Chudry N., Correa F., Silverman M. (1987). Nedocromil sodium and exercise induced asthma.. Arch Dis Child.

[PDF_00274] Comis A., Valletta E. A., Sette L., Andreoli A., Boner A. L. (1993). Comparison of nedocromil sodium and sodium cromoglycate administered by pressurized aerosol, with and without a spacer device in exercise-induced asthma in children.. Eur Respir J.

[PDF_00241] Corkey C., Mindorff C., Levison H., Newth C. (1982). Comparison of three different preparations of disodium cromoglycate in the prevention of exercise-induced bronchospasm: a double-blind study.. Am Rev Respir Dis.

[PDF_00262] Eady R. P., Greenwood B., Jackson D. M., Orr T. S., Wells E. (1985). The effect of nedocromil sodium and sodium cromoglycate on antigen-induced bronchoconstriction in the Ascaris-sensitive monkey.. Br J Pharmacol.

[PDF_00250] Eggleston P. A., Rosenthal R. R., Anderson S. A., Anderton R., Bierman C. W., Bleecker E. R., Chai H., Cropp G. J., Johnson J. D., Konig P. (1979). Guidelines for the methodology of exercise challenge testing of asthmatics. Study Group on Exercise Challenge, Bronchoprovocation Committee, American Academy of Allergy.. J Allergy Clin Immunol.

[PDF_00258] Knudson R. J., Slatin R. C., Lebowitz M. D., Burrows B. (1976). The maximal expiratory flow-volume curve. Normal standards, variability, and effects of age.. Am Rev Respir Dis.

[PDF_00271] König P., Hordvik N. L., Kreutz C. (1987). The preventive effect and duration of action of nedocromil sodium and cromolyn sodium on exercise-induced asthma (EIA) in adults.. J Allergy Clin Immunol.

[PDF_00265] Leung K. B., Flint K. C., Brostoff J., Hudspith B. N., Johnson N. M., Pearce F. L. (1986). A comparison of nedocromil sodium and sodium cromoglycate on human lung mast cells obtained by bronchoalveolar lavage and by dispersion of lung fragments.. Eur J Respir Dis Suppl.

[PDF_00246] Levison H., Reilly P. A., Worsley G. H. (1985). Spacing devices and metered-dose inhalers in childhood asthma.. J Pediatr.

[PDF_00269] Morton A. R., Ogle S. L., Fitch K. D. (1992). Effects of nedocromil sodium, cromolyn sodium, and a placebo in exercise-induced asthma.. Ann Allergy.

[PDF_00237] Patel K. R., Wall R. T. (1986). Dose-duration effect of sodium cromoglycate aerosol in exercise-induced asthma.. Eur J Respir Dis.

[PDF_00260] Silverman M., Anderson S. D. (1972). Standardization of exercise tests in asthmatic children.. Arch Dis Child.

[PDF_00254] Taussig L. M., Chernick V., Wood R., Farrell P., Mellins R. B. (1980). Standardization of lung function testing in children. Proceedings and Recommendations of the GAP Conference Committee, Cystic Fibrosis Foundation.. J Pediatr.

